# The Temporal Alignment of Speech-Accompanying Eyebrow Movement and Voice Pitch: A Study Based on Late Night Show Interviews

**DOI:** 10.3390/bs13010052

**Published:** 2023-01-06

**Authors:** Volker Gast

**Affiliations:** Department of English and American Studies, Friedrich Schiller University, 07743 Jena, Germany; volker.gast@uni-jena.de

**Keywords:** eyebrows, facial gestures, pitch, prominence, audiovisual prosody

## Abstract

Previous research has shown that eyebrow movement during speech exhibits a systematic relationship with intonation: brow raises tend to be aligned with pitch accents, typically preceding them. The present study approaches the question of temporal alignment between brow movement and intonation from a new angle. The study makes use of footage from the *Late Night Show with David Letterman*, processed with 3D facial landmark detection. Pitch is modeled as a sinusoidal function whose parameters are correlated with the maximum height of the eyebrows in a brow raise. The results confirm some previous findings on audiovisual prosody but lead to new insights as well. First, the shape of the pitch signal in a region of approx. 630 ms before the brow raise is not random and tends to display a specific shape. Second, while being less informative than the post-peak pitch, the pitch signal in the pre-peak region also exhibits correlations with the magnitude of the associated brow raises. Both of these results point to early preparatory action in the speech signal, calling into question the visual-precedes-acoustic assumption. The results are interpreted as supporting a unified view of gesture/speech co-production that regards both signals as manifestations of a single communicative act.

## 1. Introduction

### 1.1. Context of the Study: Multimodal Communication

Human face-to-face communication is multimodal, primarily relying on acoustic and visual signals [1]. The acoustic signal is produced in the vocal tract and is constituted by the segments of speech, such as [w], [ɛ], and [l] in the word *well*. The speech segments are realized with a specific prosody, i.e., melody and rhythm. For example, the word *well* can be pronounced with varying degrees of length, and different types of tone movement, e.g., falling (“Well!”) or rising (“Well?”). Prosody has various meta-propositional functions, such as the expression of emotions and attitudes [2], the indication of prominence [3], and the organisation of the conversational exchange (e.g., turn-taking) [4].

The visual signal is produced with visible body parts, most prominently the hands and the face, in the form of gestures [5,6]. There is comprehensive literature on hand gestures in the tradition established by Adam Kendon and David McNeill [7,8], covering aspects such as their structure (‘preparation’, ‘stroke’, ‘retraction’) [9,10,11], their degree of ‘autonomy’ relative to speech (from speech-accompanying gestures to the signs of sign languages [12,13,14]), and their semiotic properties (‘iconic’, ‘metaphoric’, ‘deictic’, ‘beat’ [13]). Research on facial expressions has an even longer tradition, starting with the work of Paul Ekman and Wallace Friesen in the 1960s. Ekman and Friesen investigated both the display of emotions [15,16,17] and the communicative signals [18,19,20] associated with facial expressions.

Even though there was some cross-fertilization between the study of hand gestures and facial expressions at an early stage, e.g., in the work by A. Kendon [9,10,21] and D. McNeill [13,22], it was only in the 1990s that the two types of signals started to be analyzed in tandem in a more systematic way. For example, the *Integrated Message Model of Language* proposed by Janet Bavelas, Nicole Chovil and colleagues analyze ‘conversational facial gestures’ with the toolbox developed for hand gestures [23,24,25,26,27,28,29], intending “to articulate and document the extensive similarities of facial to hand gestures, which offer an alternative to approaches that see the face as stereotypic configurations related to a few emotional expressions” [28] (p. 31).

A central, still open question of research into multimodal communication concerns the relationship between speech and visual signals from the point of view of production. The two types of signals are produced concurrently, and the question arises of whether they “share a computational stage”, in McNeill’s words [22] (p. 350)—and if so, what stage(s) they share. Put differently, the question is whether gesture and speech are two manifestations of the same action, or whether they are separate actions (see [30] for an overview of the discussion). According to the ‘separatist’ view, as expressed, for instance, in  the ‘Lexical Retrieval Theory’ [31,32,33,34], speech and gestures are processed separately, and gestures have an auxiliary role relative to, and facilitative effect on, speech. In  fact, proponents of this view hold that gestures contain very little information to begin with, and that “the gestural contribution to communication is, on the whole, negligible” [33] (p. 93). According to the unified view, often associated with McNeill’s ‘Growth Point Theory’ [22], “the speech production process is manifested in two forms of activity simultaneously: in the vocal organs and also in bodily movement” [10] (p. 211). Gestures and speech are taken to emerge from the same underlying conceptualizations, reflecting different facets of the same message [35,36,37,38,39].

One type of evidence that has been used in support of a unified view of speech-gesture co-production concerns the temporal coordination of speech and gestures. Goldin-Meadow and Brentari [40] write:

gesture and speech are temporally organized as a single system. The  prosodic organization of speech and the phrasal structure of the co-occurring gestures are coordinated so that they appear to both be produced under the guidance of a unified plan or program of action […]. For example, the gesture and the linguistic segment representing the same information as that gesture are aligned temporally. More specifically, the gesture movement—the “stroke”—lines up in time with the tonic syllable of the word with which it is semantically linked (if there is one in the sentence).

[40] (p. 16)

Advocates of a separatist view, however, point to the well-known asynchrony of gestures and speech as evidence for an early split in the production of gestures and speech. Gestures generally tend to precede the segment in the speech signal that they relate to (the ‘lexical affiliate’) [33,41,42,43]: “[t]he idea that lexical gestures have an early origin is consistent with the well-established finding that lexical gestures precede their lexical affiliates ….” [33] (p. 23).

While there is broad consensus that gestures and speech are coordinated but asynchronous, with gestures normally preceding their lexical affiliate, the details of crossmodal temporal coordination are not yet fully understood. Studying that coordination is therefore a desideratum of research into multimodal communication: “it appears that a fully comprehensive model of the speech planning process must include a mechanism that can provide an account of speech-gesture alignment in time and the relationship of both speech and gesture to structure and meaning” [44] (p. 1210). The present study intends to contribute to this agenda by investigating the temporal coordination of a prominent facial articulator, the eyebrows, and a central aspect of prosody, i.e., intonation.

### 1.2. Facial Gestures and Audiovisual Prosody

Eyebrows are associated with specific communicative functions. Four types of functions have figured prominently in research on eyebrow gestures: (i) the expression of emotions, (ii) the expression of epistemic attitudes, (iii) the expression of specific types of illocutionary acts, and (iv) the handling of turn-taking in conversation. The most prominent emotion linked to eyebrow movement is that of surprise. This association has often been noted [45,46,47,48] and is, in a way, archetypical from an evolutionary point of view. Eyebrows are raised as a result of opening one’s eyes wide, as a symptom of “attentional activity” [20,48,49,50]. Related to the expression of surprise is the epistemic attitude of uncertainty or ignorance. Ignorance is encoded in a conventionalized gesture, the ‘facial shrug’ [27,29]. Moreover, it has been shown that raised eyebrows tend to accompany modals like *may* and *might* [51]. As indicators of illocutionary force brow raises have been claimed to signal yes-no question [20,52,53,54], but this claim has also been called into question [51,55,56]. Flecha-García [55,56] found that brows tend to be raised in ‘instruct moves’ in dialogue games used for elicitation (the ‘Map task’ [57,58], e.g., “So then you go along just a few dashes” [55] (p. 68)). The role of eyebrow movement in turn-taking has been noticed in several studies [55,59,60,61], e.g., insofar as, “more frequent and longer eyebrow-raising occurred in the initial utterance of high-level discourse segments than anywhere else in the dialogue” [55] (p. ii).

Beyond the expression of communicative functions of the type mentioned above, eyebrows have been found to signal the ‘prominence’ of their affiliates in a prosodic sense. A parallelism of gestures and prosody has long been noted from the perspective of prosody [62]. D. Bolinger holds that “[t]he fluctuations of pitch are to be counted among all those bodily movements which are more or less automatic concomitants of our states and feelings and from which we can deduce the states and feelings of others. Intonation belongs more with a gesture than with grammar” [63] (p. 157). Just like the pitch trajectory moves up and down, “[o]ther up-down gestures can be carried by the eyebrows, the corners of the mouth, the arms and hands, and the shoulders. Motion in parallel with pitch is again the rule” [63] (p. 158). The assumption is that prosody and gestures cooperate in conveying prominence can be termed the *Audiovisual Prosody Hypothesis*.

Several studies have provided evidence for the Audiovisual Prosody Hypothesis using eyebrow data. Cavé et al. [64] studied the relationship between brow raises and pitch movement in elicited data. The authors manually annotated their data for brow raises, and determined the type of pitch movement at the time of the raise (rising-falling, falling-rising, plateau, rising and falling). They found an association of brow raises with contours containing a rise (rising-falling, falling-rising, and rising). These results were confirmed in a later study [59].

Flecha-García investigated (among other things) the temporal distance between brow raises and pitch accents in dialogic recordings of four subjects performing the Map Task [57,58]. With manual annotations she identified both brow raises and pitch accents, and determined distances between them. She found that the distance between brow raises and the closest pitch accent is close to zero (μ = 63 ms, σ = 458 ms), providing evidence of alignment. Moreover, brow raises “are significantly closer to their following PA [pitch accent] than to their preceding PA” [55] (p. 126). This shows that brow raises, like hand gestures, tend to precede the segment in the speech signal that they relate to.

Kim et al. [54] used elicited data from six participants to study the association of eyebrow and head movement in relation to different types of information-structural configurations (broad focus, narrow focus, echoic question). The narrow focus and echoic question conditions were associated with eyebrow action in a region of ≈250 ms preceding the prosodically prominent constituent. The tendency of brow raises to occur in the neighborhood of prominent words, and to precede those words, was thus confirmed.

Following up on Kim et al. [54], Schade and Schrumpf [65] investigated the alignment of brow raises and pitch accents, taking the additional dimension of ‘emotion’ into account. Their hypothesis was that “f0 peaks and eyebrow movement peaks [would] be temporally closer in emotional utterances” [65] (p. 95). The authors used elicited data—story retellings from an egocentric perspective—annotated with OpenFace [66] for eyebrow movement, and with Praat [67] for the audio signal. The study confirmed the results obtained by Kim et al. [54]—“pitch and eyebrow movement are temporally aligned” and showed moreover that “[e]motionality seems to enhance this alignment” [65] (p. 99).

Unlike the other studies mentioned above, Berger and Zellers [68] used naturalistic data—semi-spontaneous YouTube monologs—to investigate the interaction of intonation and eyebrow movement. On the basis of automatic OpenFace annotations and manual pitch annotations, the authors found evidence of correlations between “pitch height and eyebrow movements …for at least some of the measures for all speakers” [68] (p. 1). Moreover, they went beyond previous studies aiming to “investigate the relationships between data in the form of contours rather than individual points” [68] (p. 10). For each pitch accent, they determined eyebrow trajectories around the F0-peak in a window of 1 s. Using Functional Principal Component Analysis, the authors did not find evidence for synchronization of eyebrow peaks and F0 peaks, contradicting the findings of Flecha-García [55]. However, it is important to keep in mind that their method differed from that of Flecha-García: while Flecha-García took brow raises as a point of reference and identified pitch accents in their neighborhood, Berger and Zellers used pitch accents as a point of departure and identified brow raises in their neighborhood. The authors also point out a few caveats, e.g., that the eyebrow positions were generally rather high in the 1-s window around the pitch accents, and that cases of brows in rest position were excluded.

The present study follows up on Berger and Zellers [68] in two respects. First, it uses naturalistic, rather than elicited data. And second, it investigates contours, in addition to point measurements. Pitch is mostly studied in one of two ways, in the field of phonology. It can either be regarded as being constituted by pitch trajectories (rise, falls, rise-falls …), or by pitch levels or targets (high, low). The first approach is typically associated with what is often called the ‘British school’ of intonation [69]; the second one, particularly the ‘autosegmental’ paradigm, has its roots in the US and is therefore often called the ‘American school’ [70]. The present study takes the perspective of the British school of intonation and thus treats pitch as a dynamic, rather than positional, signal. This has consequences for the question of temporal alignment between eyebrow positions and pitch. Eyebrow positions can reasonably be assumed to constitute a positional signal: a high position is associated with a high degree of prominence. This is different for pitch: prominence is not associated with a particularly high fundamental frequency, but with a high degree of change in the signal. From this point of view, we would wish to correlate eyebrow positions with properties of pitch contours, rather than point measurements.

There are important differences between the present study and the one by Berger and Zellers. First, the data is dialogic, as in most of the other previous studies mentioned above. As Berger and Zellers [68] note, eyebrow movement in YouTube monologs may not be entirely natural. Second, the study is completely data-driven, using no manual annotations, and thus not relying on any theoretical preconceptions (which may be controversial in the domain of phonology). Unlike Berger and Zellers [68], and like Flecha-García [55], the brow raises are moreover taken as a point of reference, and pitch movement is investigated in relation to brow raises. The reason is that—as pointed out above—eyebrow height is a (positional) point measurement, with the highest measurement corresponding to the highest visual prominence level, whereas pitch contours are regarded as dynamic signals whose prominence is reflected in degrees of change, not absolute values. Finally, the present study differs from that of Berger and Zellers [68] in that it refrains from using high-level, and potentially black-box, methods of analysis, remaining close to the ‘observable’ signal. Pitch contours are treated as near sinusoidal functions. The parameters of these functions—specifically the amplitude and the ordinary frequency—are subject to constant change, but relatively stable within individual cycles (up-and-down movements). The parameters of the sinusoidal functions fitted to the data are regarded as correlates of ‘dynamicity’, and are used as input to the quantitative analysis.

### 1.3. Objectives, Significance and Structure of the Study

The present study is intended as a contribution to our understanding of the relationship and interaction between prosody in the speech signal and eyebrow movement as a prominent facial articulator. Based on the Audiovisual Prosody Hypothesis, which holds that acoustic and visual signals jointly convey prominence, it studies pitch movement in the neighborhood of brow raises. Importantly, the pitch is not only treated as a series of point measurements, but also as a sinusoidal function. This approach allows us to determine not only levels of pitch, but also degrees of dynamicity (amplitude and frequency of the signal), and the shape of the signal.

The results confirm observations made in previous studies, specifically concerning the temporal alignment of brow raises and pitch. The data show that brow raises tend to be followed by higher-than-average pitch values. Moreover, the post-peak region shows more fluctuations in the signal, i.e., higher degrees of dynamicity.

The study also arrives at new results. The shape of the pitch contour in the pre-peak region correlates with the height of the brow raise. In a window of approx. 630 ms before the brow raise, a later start in the phase of a sine wave–with the pitch falling, or rising from a low level–correlates with higher brow peaks. This finding has potential implications for the co-production of brow raises and prosody. While it is generally assumed that the visual signal (the brow raise) tends to precede the acoustic signal (the pitch accent), the shape of the pitch contour in the pre-peak region points to a ‘preparatory’ phase before the actual prominence marking, and can be regarded as an early symptom of prominence. The observation of a substantial preparatory phase in the pitch contour sheds doubt on the visual-precedes-acoustic assumption.

The study is structured as follows: Section 2 contains a description of the data and the methods of analysis. Section 3 presents the results, which are discussed in Section 4.

## 2. Materials and Methods

### 2.1. The Corpus and the Processing of the Data

The present study is based on footage from the *Late Night Show with David Letterman* recorded between 1980 and 2014. The material was obtained from a fan page (https://donzblog.home.blog/, accessed on 29 December 2022). The clips come with automatically generated subtitles (in srt-format), with a reasonable, though not perfect, quality. The corpus comprises 160 files, with some files covering several shows. Altogether, the corpus contains approx. 160 h of recording. Obviously, not all of the material can be used for the analysis of facial gestures in relation to prosody, as the camera angles vary, there is overlapping speech (introducing noise into the audio signal) and sometimes, the camera focuses on the guest when the host speaks. A sample of video sequences was therefore created manually, as detailed below.

**Figure 1:**
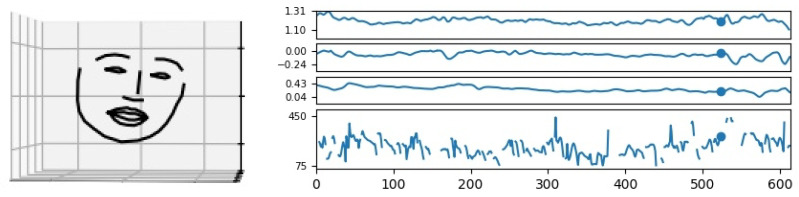
A frame with 3D facial landmark annotation (left) and measurements of eyebrow height, sagittal head angle, lateral head angle, and pitch; the segment has been taken from Episode 0148 featuring Art Donovan. Original frames are not shown for reasons of copyright.

In the first step, faces were recognized in the video signal, using the dlib-package for Python (https://pypi.org/project/dlib/, accessed on 29 December 2022). Only sequences of frames were used that contained at least three seconds of recording of the same face, from the same angle, at a resolution of at least 10,000 pixels per face. The video data was processed at a rate of 30 frames per second. All measurements are associated with frames, and 30 subsequent measurements correspond to one second of recording. The faces thus recognized were annotated for facial landmarks using 3D facial landmark detection software [71] (https://github.com/1adrianb/face-alignment, accessed on 29 December 2022). Thefacial landmarks delivered by this package are comparable to those of OpenFace (https://github.com/TadasBaltrusaitis/OpenFace, accessed on 29 December 2022), though no Action Units were determined, in keeping with the data-driven approach of the study. The software identifies 68 facial landmarks, which can be used to measure out the face of the speakers, and to create a model of the speaker’s face, see Figure 1 on the left for an example.

The facial landmark annotations were used to obtain measurements for three variables relevant to the study of facial gestures: (i) the height of the eyebrows, (ii) the lateral angle of the head, and (iii) the sagittal angle of the head (the calculation of the angles is only possible with 3D models). The height of the eyebrows was measured as the ratio of the distance between the centroid of the four landmarks delimiting the nose, and the centroid of the ten points marking the eyebrows. The measurements were standardized by a speaker.

The acoustic signal was processed with Praat [67], at a rate of 30 measurements per second, like the video data. The data thus obtained was visualized for manual inspection as shown on the right side of Figure 1. The 3D-model of the speaker is shown on the left, and the four signals of interest are displayed on the right (from top to bottom, eyebrow height, lateral head angle, sagittal head angle, and pitch; the measurements are contained in the file df_measurements.tsv in the Supplementary Materials).

### 2.2. The Sample Used for the Analysis

The corpus was partitioned into *segments* corresponding to subtitle units as represented in the automatically generated subtitle files. This way of partitioning the data allowed us to control the lexical material associated with the pitch and eyebrow data, which proved particularly useful when it came to manually inspecting and searching the corpus. We regard the segments as a random sample of sequences from the corpus, with mean durations of 2.28 s (σ=1.08). A sequence of such segments is shown in (1) and can be inspected by clicking 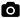. The segments are identified by the number of the ‘episode’ (mp4-file) and the subtitling segment (e.g., 0145-2372 for segment 2372 in the subtitle file 0145.srt)

(1)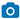 
  - …this movie and the same guy did my            [0145-2372]
  - makeup for coming to America Rick Baker      [0145-2373]
  - he turned me into this 400 yeah he                    [0145-2374]
  - turned me to this 400-pound guy and uh         [0145-2375]

To control for speaker-specific effects, a sub-sample was created—the *speaker sample* in the following—with data from five speakers who figure prominently in the show (as recurrent guests), and whose facial landmarks did not show any systematic errors: Art Donovan (AD), Buck Henry (BH), Eddie Murphy (EM), Teri Garr (TG), and Quentin Crisp (QC). The sample was manually inspected to make sure that the interviewee, who is filmed, speaks while being filmed. The sample thus created consists of 887 segments, with 55,504 frames and 1850 s (AD: 339 segments, 20,552 frames, 685.1 s; BH: 97 segments, 6972 frames, 232.4 s; EM: 151 segments, 8385 frames, 279.5 s; QC: 11 segments, 9180 frames, 306.0 s; TG: 187 segments, 10,415 frames, 9180 frames, 347.2 s; the measurements for the sample are contained in the file df_sample.tsv in the Supplementary Materials).

For the study of pitch contours in the environment of brow raises the data was processed as follows: we identified in each segment the position where eyebrow height was at its maximum, the ‘eyebrow peak’. 120 measurements around the eyebrow peak were extracted, up to 60 pitch measurements preceding it and up to 60 measurements following it. This gave us, for each corpus segment, a series of 121 measurements (as the pitch and eyebrow height at the peaks were also included), and a window of 2s around the eyebrow peak (the pitch measurements around the eyebrow peak are contained in the file df_sample_pitch.tsv, and  the corresponding eyebrow measurements are contained in the file df_sample_eb.tsv in the Supplementary Materials).

**Figure 2:**
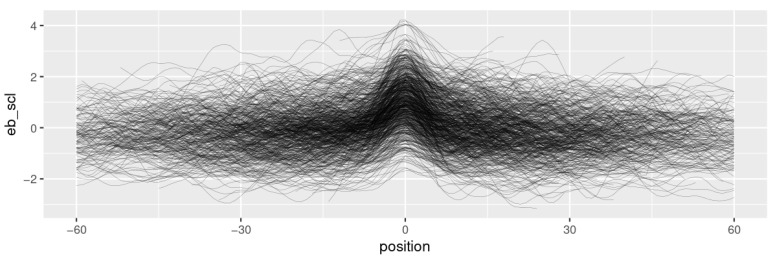
Time series for eyebrow movement around the peak of a segment (speaker sample). The values are scaled per speaker.

**Figure A1:**
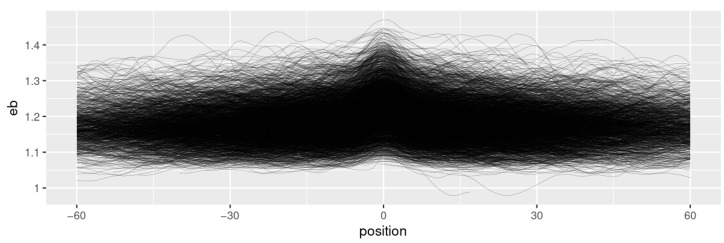
Time series for eyebrow movement around the peak of a segment (complete sample). The values are scaled per segment.

Figure 2 shows the series of eyebrow measurements for the speaker sample. The raises exhibit comparable widths, covering approx. a period of ten to twenty frames around the peak (≈300–600 ms). The raises seem to be largely symmetrical. A plot showing the eyebrow series for the entire dataset is provided in the Appendix A (Figure A1). According to a Mann-Whitney U test, the eyebrow heights between positions −8 and 9 (in a window of approx. 270–300 ms around the peak) are significantly higher than the mean. I assume that this window approximately corresponds to the scope of the brow raise.

### 2.3. Fitting Sinusoidal Models to the Data

**Figure 3:**
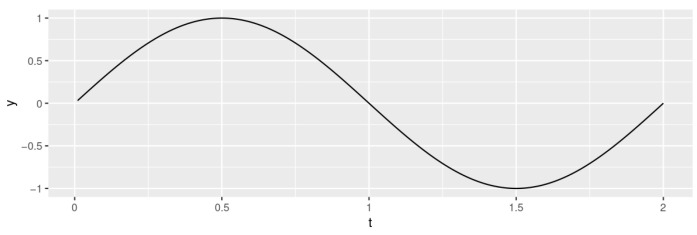
Sine wave, with phases on x-axis.

To detect correlations between eyebrow height and pitch measurements, sinusoidal models were fitted to windows of different lengths to the data preceding the eyebrow peak (the ‘pre-peak region’) and the data following it (the ‘post-peak region’), filling in missing values with linear interpolation. While pitch does not generally follow a sinusoidal pattern, the individual ups and downs characterizing the tones of English can be captured with such a model. A sine wave is standardly described in terms of three parameters: (i) the amplitude (*A*), (ii) the ordinary frequency (*f*), (iii) and the phase (ϕ). The amplitude corresponds to the difference between the center of the wave and the absolute values of its highest and lowest points. The ordinary frequency represents the number of cycles per time unit, and thus the rate of pitch changes. The phase ϕ, measured in radians, corresponds to the part of the cycle where the waveform starts, and ranges from 0 to 2. I will refer to the phases as ‘high-rising’ (0≤ϕ<0.5), ‘high-falling’ (0.5≤ϕ<1), ‘low-falling’ (1≤ϕ<1.5) and ‘low-rising’ (1.5≤ϕ<2). Figure 3 illustrates a sine wave with the ϕ-value (for the phase) on the x-axis.

In addition to the standard parameters of a sine wave we included a y-intercept, i.e., a value shifting the entire curve up or down on the y-axis. This intercept (*I*) will be called the ‘baseline shift’. (2) shows the model equation for the sine wave (*t* is a point in time). The models were fitted using the function nls() of R [72] (the parameters for the best-fitting curves are contained in the files res_df_nls_pre.tsv for the pre-peak region, and  res_df_nls_post.tsv for the post-peak region.)

(2)
y=I+A×sin(2×π×f×t+ϕ×π)

**Figure 4:**
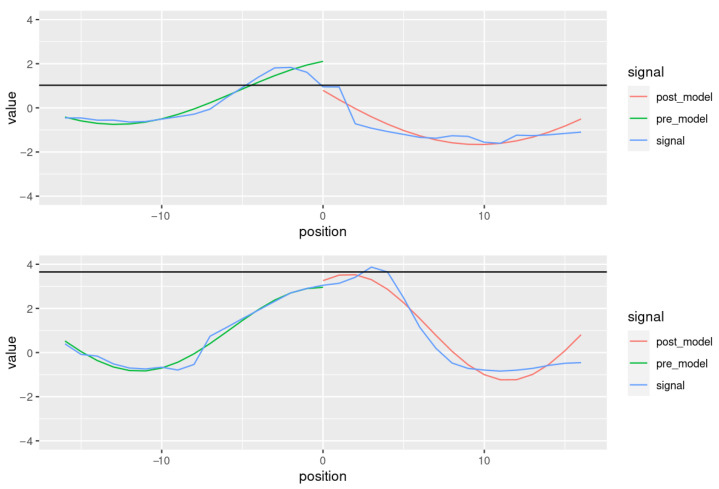
Two pitch contours with sinusoidal fittings (segments 0099-0249 and 0099-0337, both by QC). The blue line shows the pitch contour, the green line shows the sine curve fitted to the pre-peak region, and the red line shows the sine curve fitted to the post-peak region.

The sinusoidal models fitted to the pre- and post-peak regions of two segments are shown in Figure 4, for a window size of 17 frames (≈570 ms). The blue lines show the pitch measurements, the green lines represent the sinusoidal model fitted to the 16 measurements preceding the eyebrow peak as well as the peak, and the red line shows the model fitted to the pitch contour following the peak. The black line shows the value of the eyebrow peak (in standard deviations per speaker). The post-peak model in the upper plot shows a sine wave with an amplitude of 3.3, an ordinary frequency of 0.37, and a ϕ-value of 1.04 (i.e., it starts in the early low-falling phase). The post-peak model in the bottom plot shows a sine wave with an amplitude of 2.4, an ordinary frequency of 0.86, and a ϕ-value of 0.24 (it starts in the late high-rising phase). The contour in the bottom plot is more dynamic than the one in the upper plot insofar as both the range (*A*) and rate (*f*) of pitch values is higher.

In the statistical analysis, the parameters determined for each pre- and post-peak contour were used as predictors for the eyebrow height at its peak, using mixed regression modeling, with ‘speaker’ as a random effect (intercept only).

## 3. Results

### 3.1. Correlations between Pitch and Eyebrow Height Measurements

**Figure 5:**
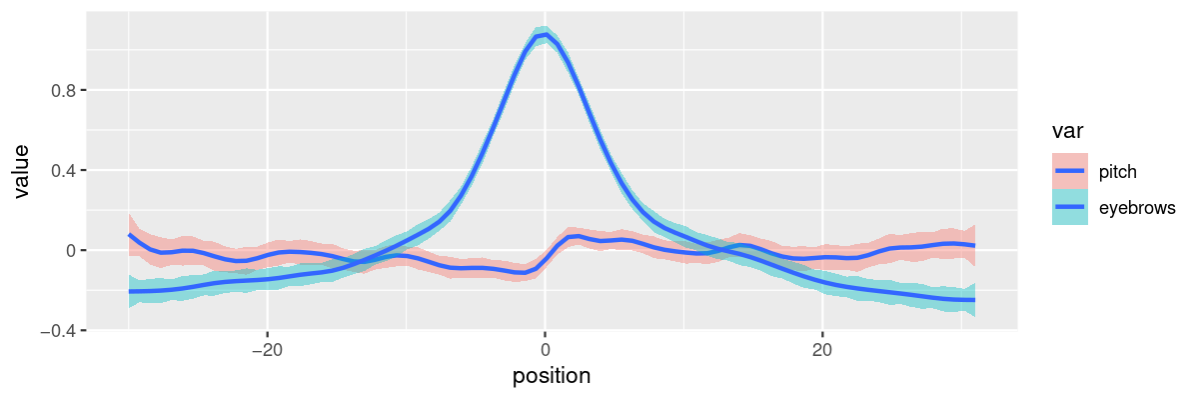
Smoothed curves showing the eyebrow height (blue) and pitch value (red) of the speaker sample, with 95% confidence intervals for the standard errors. The smoothing curves show a generalized additive model fitted to the data (k = 30).

Figure 5 shows a smoothed curve (generalized additive model, k = 30) representing the eyebrow height (blue line) and pitch values (both standardized per speaker) with 95% confidence intervals for the standard error. The pattern is remarkably clear. Eyebrow movement is symmetrical (see also Figure 2), while the pitch curve drops before the eyebrow peak, then raises, crossing the zero line approximately at the peak, and remains at a higher-than-average level for a few frames.

**Figure 6:**
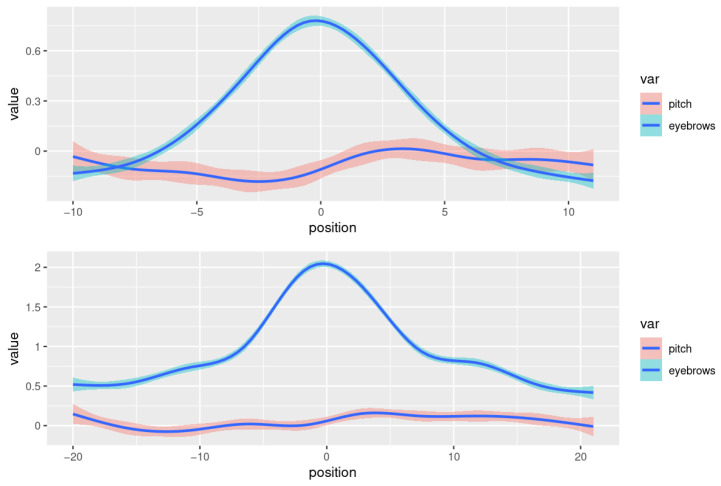
Smoothed curves showing the eyebrow height (blue) and pitch value (red) of the speaker sample, with 95% confidence intervals for the standard errors, for segments with an eyebrow peak of <1.5 standard deviations (top plot) and an eyebrow peak of >1.5 standard deviations (bottom plot). The smoothing curves show generalized additive models fitted to the data (k = 10).

Figure 5 aggregates all types of brow raises—very low ones, which are likely not intended as facial gestures but purely co-articulatory, and high ones, which (may) convey a meaning of their own. Figure 6 shows the curves for eyebrow peaks with a height of <1.5 standard deviations (top plot), and those with a peak of >1.5 standard deviations (bottom plot). The low raises show a pattern where the pitch is well beneath average and rises to reach an average height shortly after the eyebrow peak. In the neighborhood of the higher raises, the pitch is at an average level before the eyebrow peak and raises from there to a higher-than-average value for a certain period. The generalized additive model (with k = 10) shows the lower bound of the standard error to be >0 for positions −1 to 19. This confirms the results obtained in studies showing that pitch tends to be elevated in the region following a (gestural) brow raise.

### 3.2. Correlations between Eyebrow Measurements and the Parameters of Sinusoidal Functions

**Figure A2:**
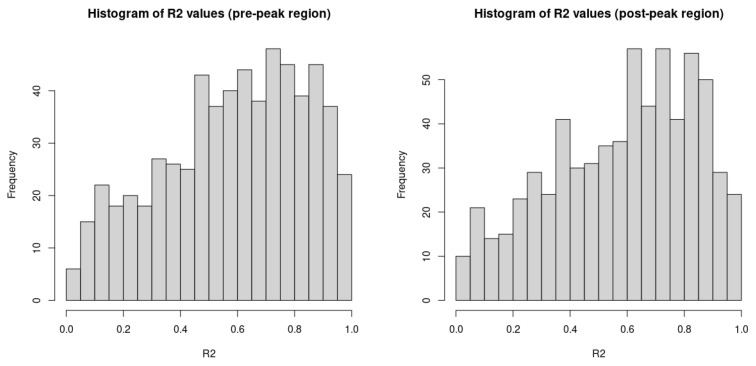
Histograms for the r-squared values of the models fitted to the pre-peak region (**left**) and the post-peak region (**right**), with a window size of 20 frames.

The sinusoidal models fitted to the pitch contours generally show a rather good fit, with the coefficients of determination averaging at $r^{2}=0.58$, for both the pre- and post-peak regions, for a window size of 20. Histograms for the $r^{2}$-values of these models are shown in Figure A2 in the Appendix A. R^2^-values do not show any correlations with other variables used for the analysis, i.e., the goodness of fit does not vary with the type of contour.

Mixed-effects models (with a random intercept for ‘speaker’) were fitted to pitch series of different window sizes, from 10 to 20 frames. The parameters of the sinusoidal functions fitted to the pre-peak region show significant correlations in the larger range of window sizes. The best model was obtained at a window size of 19 frames.

Table 1 shows the estimates for the main effects of this model, with the standard errors and the results of a t-test, as delivered by the lmer()-function of the lmerTest-package for R [73]. The parameters for amplitude (*A*) and ordinary frequency (*f*) show a positive correlation with the value of the eyebrow height at its peak. Moreover, the phase parameter ϕ shows a positive correlation with the maximum eyebrow height. This means that the contours accompanying higher brow raises tend to start in the later phases of a sine wave. There is no correlation between the maximum eyebrow height and the baseline shift. No interactions between the parameters were found.

**Figure 7:**
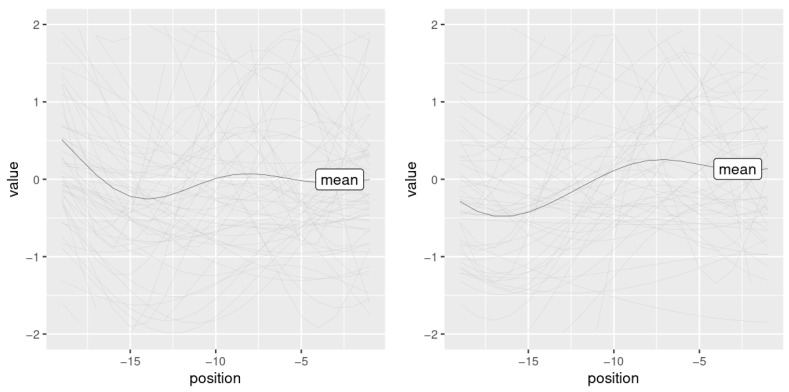
Fitted sinusoidal curves for the pre-peak regions, within a window of 19 frames. The thick lines in the center show the mean values.

For illustration, consider Figure 7, which shows sinusoidal models fitted to the pre-peak region at a window size of 19 frames that start with a high-falling (left) and a low-falling contour (right). The plot only shows pitch trajectories of segments with an eyebrow peak of >1.5. It is this type of contour that tends to accompany higher brow peaks.

The best model fitted to the post-peak region was based on the parameters of models fitted to a window of 17 frames. The linear model shows similar—in fact, stronger—effects for amplitude (*A*) and ordinary frequency (*f*) than the pre-peak model, but the phase has no significant effect. As in the case of the pre-peak region, there is no correlation with the baseline shift. Again, no interactions were found. The estimates of a model with significant predictors only are shown in Table 2.

To compare the correlations between the pre- and post-peak phase with the height of the eyebrow peak, we fitted a model with variables from both regions, with the parameters for the window sizes providing the best models for either region (19 frames for the pre-peak region and 17 frames for the post-peak region). The model includes all those variables that have a significant effect in the separate models for the pre- and post-peak regions. Table 3 shows the estimates.

In a combined model, the amplitude of the pre-region does not show a significant effect (*p* = 0.56). The ordinary frequency of the pre-peak region exhibits a tendency, but is not significant at α = 0.05 (*p* = 0.07). The ϕ-value of the pre-peak region remains significant (p=0.02). The effects for amplitude and ordinary frequency of the post-peak region show similar magnitudes and levels of significance as in the model fitted to the post-region only.

## 4. Discussion and Conclusions

Previous studies have shown that brow raises typically (though not necessarily) occur in the vicinity of pitch accents, either synchronous with or preceding them. This tendency has been confirmed by the data analyzed in the present study. Pitch values tend to be higher than average in a region of approx. 670 ms following the peak of the brow raise in those cases where the eyebrow peak has a height exceeding 1.5 standard deviations. This seems to show that the visual signal (the brow raises) does in fact precede the acoustic signal (pitch).

The results obtained on the basis of absolute pitch measurements have to be interpreted with a grain of salt, however. It seems reasonable to use positional measurements for the height of the eyebrows, as operationalizations of visual prominence: higher brows convey more prominence than lower brows. By contrast, in the acoustic signal prominence is conveyed through the degree and rate of change, not through absolute position. This means that to understand the temporal alignment between the two signals, we need to compare degrees and rates of change in the acoustic signal with absolute values in the visual signal.

In the present study the dynamic nature of the pitch signal was taken into account by not comparing positional values of eyebrow positions with absolute pitch measurements, but with properties of the pitch contours as reflected in the parameters of sinusoidal models fitted to the data. In this way we obtained measurements of the degree of ‘dynamicity’ of a contour (amplitude/*A* and ordinary frequency/*f*), and its shape (as reflected in the phase ϕ).

The results show that the amplitudes and ordinary frequencies in the post-peak region exhibit a positive correlation with the maximum height of the eyebrows in a brow raise. No preference for a specific shape could be identified in this region. A model fitted to the pre-peak region shows similar effects as far as dynamicity is concerned. However, the effects of amplitude and ordinary frequency disappear in a combined model with the post-peak region. The pre-peak values for *A* and *f* are less informative than the post-peak values, as reflected in their lower regression coefficients, and they do not provide additional information in comparison to the post-peak values. Still, it should be borne in mind that when considered on their own, they do show significant correlations with the eyebrow height at its peak.

Perhaps the most interesting finding about the pre-peak region is that the shape of the signal is informative. Within a window of 19 frames preceding the peak, there is a significant tendency for the contours to start at a later stage in the cycle of a sine wave. This means that the pitch is either falling, or rising from a low position. The fact that the shape of the pitch contour at that stage is not random points to a preparatory phase in the expression of prominence. The exact contours need to be investigated further, with attention to more potential predictors, such as the lexical material in the context of the brow raise.

As for the more general question concerning the relationship between visual and acoustic signals, the present study confirms the ‘visual-first’ tendency observed in earlier studies. However, there are ‘early symptoms’ of prominence—and, hence, temporal alignment—as well. The period at which the shape of the contour is informative, relative to the position of the eyebrows at the peak, spans approx. 630 ms and thus precedes the onset of the brow raise, which (typically) starts approx. 270 ms before the peak.

The early symptoms of prominence in the acoustic signal may indicate that the pitch contour has a relatively long planning phase, in comparison to the eyebrow movement. The relative lateness of the perceptible symptoms of prominence in the acoustic signal could thus be due to the fact that pitch cannot be changed instantly, as it constitutes a time series with auto-correlations. Pitch forms structured, near-sinusoidal signals with regular and rule-governed ups and downs, and prominence marking needs to be integrated into that signal, which requires adaptations spanning a certain period of time, potentially leading to a lag in comparison to the visual signal.

With respect to the question of the “computational stages” shared by gestures and speech [22], the results of the present study are compatible with a one-action view of gesture/speech co-production. In particular, the degree of asynchronicity of the two signals has been relativized. The early symptoms of prominence in the acoustic signal point to a preparatory phase of ≈270 ms preceding the brow raise. While the two signals are not perfectly aligned, they seem to be co-generated in a systematic and structured way, following a “unified plan or program of action” [40] (p. 16).

## Figures and Tables

**Table 1:** Estimates for main effects of a mixed linear model predicting the magnitude of the eyebrow peak in a segment based on the parameters of the sinusoidal function for the pre-peak region, with a window size of 19 frames (≈630 ms).

	Estimate	Std. Error	df	t Value	Pr(>|t|)
Intercept	0.645	0.122	82.693	5.270	<0.001
$A_{pre}$	0.043	0.021	609.598	2.027	0.043
$f_{pre}$	0.240	0.090	609.332	2.675	0.008
$\phi_{pre}$	0.197	0.078	609.991	2.538	0.011

**Table 2:** Estimates for main effects of a mixed linear model predicting the magnitude of the eyebrow peak based on the parameters of the sinusoidal function for the post-peak region, with a window size of 17 frames (≈570 ms).

	Estimate	Std. Error	df	t Value	Pr(>|t|)
Intercept	0.633	0.120	38.261	5.254	<0.001
$A_{post}$	0.153	0.049	658.456	3.149	0.002
$f_{post}$	0.349	0.090	658.474	3.885	<0.001

**Table 3:** Estimates for main effects of a mixed linear model predicting the magnitude of the eyebrow peak based on the parameters of the sinusoidal function for the pre-peak region, with a window size of 19 frames (≈630 ms), as well as the post-peak region, with a window size of 17 frames (≈570 ms).

	Estimate	Std. Error	df	t Value	Pr(>|t|)
Intercept	0.213	0.210	210.104	1.012	0.313
$A_{pre}$	0.038	0.065	397.280	0.584	0.560
$f_{pre}$	0.217	0.120	396.176	1.817	0.070
$A_{post}$	0.162	0.059	396.820	2.738	0.006
$f_{post}$	0.387	0.110	397.808	3.523	<0.001
$\phi_{pre}$	0.238	0.101	397.285	2.360	0.019

## Data Availability

The original footage is copyright protected but can be inspected at https://donzblog.home.blog/, accessed on 29 December 2022. The relevant measurements are made available in the Supplementary Materials.

## Supplementary Materials

The Supplementary Material with the data can be downloaded at: https://doi.org/10.5281/zenodo.7485020.
